# Impact of the 340B Pharmacy Program on Services and Supports for Persons Served by Hemophilia Treatment Centers in the United States

**DOI:** 10.1007/s10995-018-2545-7

**Published:** 2018-06-12

**Authors:** Rebecca A. Malouin, Laurel Mckernan, Ann Forsberg, Dunlei Cheng, John Drake, Kathryn McLaughlin, Marisela Trujillo

**Affiliations:** 1 Department of Family and Community Medicine, Michigan State University, 909 Fee Road, Room B201, East Lansing, MI 49923 USA; 2 Comprehensive Hemophilia and Thrombosis Center, Dartmouth-Hitchcock Medical Center, 1 Medical Center Drive, Lebanon, NH 03756 USA; 3 National Hemophilia Program Coordinating Center, American Thrombosis and Hemostasis Network, 72 Treasure Lane, Riverwoods, ILL 00615 USA; 4 American Thrombosis and Hemostasis Network, 72 Treasure Lane, Riverwoods, ILL 00615 USA; 5 Great Plains Regional Hemophilia Network, Gulf States Hemophilia and Thrombophilia Center, University of Texas Health Science Center at Houston, 6655 Travis, Suite 400, Houston, TX USA; 6 Genetic Services Branch, Maternal and Child Health Bureau, Health Resources and Services Administration, Parklawn Building, Room 18A-19, 5600 Fishers Lane, Rockville, MD 20857 USA; 7 Gulf States Hemophilia and Thrombophilia Center, University of Texas Health Science Center Houston, 6655 Travis St., Suite 400, Houston, TX 77030 USA

**Keywords:** 340B Drug Pricing Program, Hemophilia, Hemophilia treatment centers, Comprehensive care model, Children with special healthcare needs

## Abstract

*Purpose* Hemophilia Treatment Centers (HTCs) provide integrated and comprehensive services to individuals affected with rare bleeding disorders, such as hemophilia and Von Willebrand disease. Through the 340 Drug Pricing Program, HTCs may use pharmacy income to support clinical staff and patient services. The objective of this study was to describe the impact of the 340B program funding on services and support provided by HTCs to persons affected by rare bleeding disorders. *Description* Federally designated comprehensive HTCs with established 340B programs were invited to participate in a mailed survey in 2014. Participants were requested to report on 340B program-funded staff and services in the calendar year 2013. *Assessment* The 31 of 37 HTCs responding served over 10,000 individuals, or one-third of the national HTC patient population. The majority of responding HTCs reported that 340B program income supported over 90% of staff such as nurses, social workers, and physical therapists. *Conclusion* The results from this survey of 31 centers with established programs demonstrates the HTCs’ reliance on 340B program support for vital comprehensive services, that are otherwise non-reimbursable, and highlights the importance of the 340B program in sustaining the high quality of care and in increasing access for a geographically dispersed, medically vulnerable population.

## Significance

*What is already known on this subject?* Hemophilia Treatment Centers (HTCs) provide comprehensive services to a medically vulnerable population affected by rare bleeding disorders. HTCs are eligible as covered entities to use pharmacy income from the 340B Drug Pricing Program to support clinical staff and patient services.

*What this study adds?* This study provides new information on the impact of the 340B Drug Pricing Program in supporting the comprehensive care model, the evidence-based standard of care for persons affected by rare bleeding disorders, within the HTCs. Income generated through the 340B Drug Pricing Program supports most of the multidisciplinary staff as well as care coordination, community education, and other support activities provided by the HTCs.

## Background

Since 1975, the Health Resources and Services Administration (HRSA) has funded a national network of 135 Hemophilia Treatment Centers (HTCs) to provide integrated services to and increased access to care for children and adults with rare, inherited bleeding disorders (Baker et al. 2005; Grosse et al. 2009). These HTCs currently provide services to over 30,000 individuals affected by hemophilia, von Willebrand disease (VWD), and other rare coagulation disorders. Hemophilia, the best-known inherited coagulation disorder, is characterized by a deficiency in clotting factor VIII (Hemophilia A) or clotting factor IX (Hemophilia B) (Srivastava et al. 2013) (Srivastava et al. 2013). Over 21,000 persons with hemophilia are treated in the HTC network, representing approximately 67% of the 31,000 affected by hemophilia in the United States (Soucie et al. 1998). Clinical symptoms include spontaneous bleeding into joints that results in degenerative joint disease if not treated appropriately and quickly with clotting factor. Spontaneous bleeding into the neck or head can be life threatening to affected individuals. Patients are encouraged to independently manage bleeding by intravenous self-treatments. By treating bleeding quickly, painful and costly complications, resulting in emergency treatment, can be avoided. The average annual cost of treating individuals affected with mild hemophilia, without complications, is approximately $59,101 per patient and $301,392 for patients with severe hemophilia receiving prophylactic treatment (Zhou et al. 2015).

VWD affects both men and women and is characterized by a qualitative or quantitative deficiency of von Willebrand factor, a protein that is required for platelet adhesion. VWD is characterized by prolonged bleeding following trauma or during menstruation (Sadler et al. 2000). It is estimated that VWD affects up to 1% of the general population or over 3 million individuals (Rodeghiero et al. 1987; Werner et al. 1993). The prevalence of other inherited coagulation disorders is unknown. Inherited bleeding disorders are complicated and expensive to treat and optimal care requires a multidisciplinary team with expertise in chronic care management of these disorders.

HTCs are composed of a multidisciplinary core team including a pediatric or adult hematologist, a nurse coordinator, a social worker and a physical therapist. Services of other providers including orthopedists, dentists, genetic counselors, dieticians, and financial counselors are either provided at or coordinated through the HTC (Ruiz-Saez 2012; Yeung et al. 2016). The annual comprehensive care clinic, an annual comprehensive assessment of a patient, is an essential component of the comprehensive care model. The comprehensive care visit involves collaborative development of a care plan between the core team, the patient, and the patient’s family. In addition to the comprehensive care clinic, various services are offered to patients between their visits, including follow-up outpatient visits to assess specific issues, coordination of procedures, education to schools, teaching of self-infusion skills, and offering of psychosocial support. The goal of this integrated care model is to foster self-management and support within the patient’s community (Hoots 2003). Individuals with bleeding disorders treated within HTCs have reduced morbidity and mortality compared to those not receiving treatment in HTCs (Soucie et al. 2000; Yeung et al. 2016). Early studies found significant cost savings to both the individual and society following implementation of the comprehensive care model (Smith et al. 1982; Smith and Levine 1984).

In 1992 Congress established the 340B Drug Pricing Program (340B program) as part of the Public Health Service Act which allows designated covered entities, including HTCs, to purchase pharmaceutical products at a discounted rate (340B Drug Pricing Program 2017). Through this program, HTCs can establish a pharmacy to purchase and sell clotting factor and other drugs used by their patients to treat their bleeding disorders. HTCs can thereby augment scarce federal resources and generate program income. The program income generated by the sale of the products, by law, must be used for patient health, education, and supportive services necessary to provide comprehensive care to patients served by the HTCs (340B Drug Pricing Program 2017). Much of this program income is used to support personnel. In 2014, 83 HTCs reported to the Health Resources and Services Administration that they supported 569 full-time equivalent (FTE) positions through their 340B program income (National Hemophilia Program Coordinating Center 340B Pharmacy Program Income Survey Summary 2016). Since many staff members are only partially funded by the 340B program, the number of FTEs reported significantly underrepresents the total number of staff supported by the program. The objective of this study was to describe the impact of the 340B program funding on services and support provided by HTCs to persons affected by rare bleeding disorders.

## Methodology

**Fig. 1:**
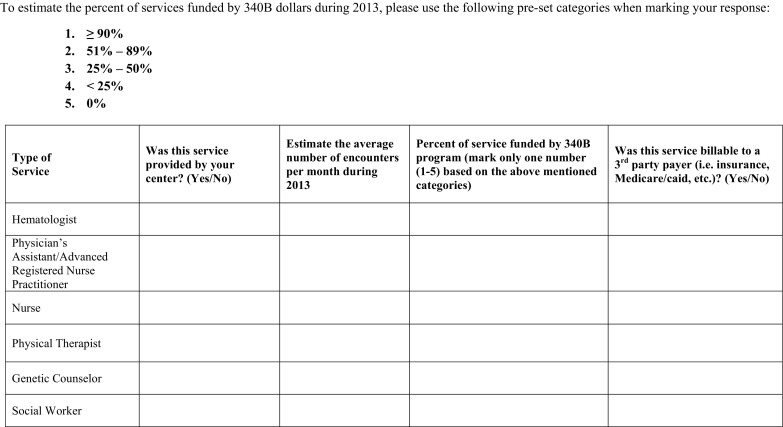
Example from Questionnaire Regarding Comprehensive Care Visit Services. To estimate the percent of services funded by 340B dollars during 2013, please use the following pre-set categories when marking your response

Staff and consumers participating in 340B programs developed a questionnaire to collect data on the use of 340B program income by HTCs. The questionnaire included three demographic questions related to the HTC and five additional tables of questions organized by types of services offered by the HTC. The services were categorized as comprehensive clinic visit services; in-person outpatient and follow-up services; coordination of care and case management; patient/family education, support and community provider support, and camps for children and their families; and comprehensive care services provided through outreach and satellite clinics, and telemedicine. The comprehensive care visit services and the in-person outpatient and follow-up services included physician, nursing, social work, physical therapy, and genetic counseling services. Services within coordination of care and case management included telephone triage/urgent care, medical care coordination and care management, and psychosocial and vocational services. Services within patient/family education and support included patient education, community provider education, peer support, and financial assistance with transportation. The questionnaire included questions on the range, utilization, and percentage of 340B funding of services offered as exemplified in Fig. 1.

Federally designated comprehensive HTCs with established 340B programs, defined as programs capable of generating sufficient 340B program income to support services, were identified through the Hemophilia Alliance website and informed about the study through presentations at national 340B member organization meetings (Hemophilia Alliance 2018). Thirty-seven out of 103 HTCs (36%) with a 340B program were invited to participate and sent the questionnaire via email. HTC size and geographic diversity were taken into consideration during recruitment through purposeful sampling. In an effort to identify the breadth of services offered at HTCs, investigators employed purposeful sampling of centers with over 250 patients representing each of the 8 HRSA regional hemophilia network regions (National Hemophilia Program Coordinating Center 2018). The questionnaire was requested to be completed by the individual responsible for the management of the 340B Program at the HTC. The survey was administered in 2014 and participants were requested to report on 340B program-funded staff and services in the calendar year 2013. Data were aggregated and univariate descriptive analyses were conducted using IBM SPSS Statistics 24. The research was conducted in accordance with prevailing ethical principles. As the unit of observation was the HTC rather than human subjects, the research was not reviewed by an institutional review board.

## Results

A total of 31 of 37 questionnaires were completed and returned, resulting in an 84% response rate. The majority of the centers (87%) reported a patient population size of greater than 250 patients, with 42% reporting greater than 400 patients. As only 22% of the 103 HTCS with 340B programs had greater than 350 patients in 2013, larger HTCs are over-represented in the sample. Nineteen of the 31 respondents were described as a university academic institution, 4 were within a children’s hospital and 8 were free standing treatment centers.

Applying the mean number of patients in each HTC size category (500 was used for the > 400 category) multiplied by the number of HTCS in that category, the total estimated number of patients served by the centers responding to this survey is over 10,000 individuals. All responding HTCs reported serving patients diagnosed with hemophilia, VWD, other factor deficiencies, and platelet disorders. Not all of the HTCs responded to every question, but all HTCs responded to the majority of questions.

### In-Person Comprehensive and Outpatient Services

**Fig. 2:**
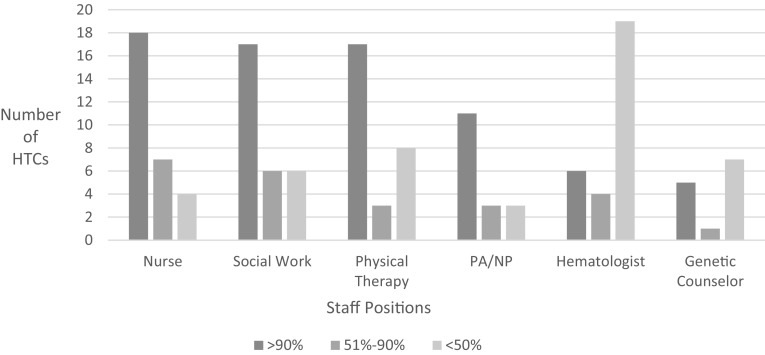
Number of HTCs reporting clinical staff position funded by 340B program. 29 of 31 HTCs responded to these questions

Twenty-nine out of 31 respondents (94%) reported they provided the four core comprehensive care services (physician, nursing, social work, and physical therapy services) at clinic visits in 2013. All 31 respondents indicated they utilized 340B program income to support the salaries of their core staff and other staff. As shown in Fig. 2, the majority of centers rely on 340B program income to fund > 90% of the staff time of nurses, social workers and physical therapists (Fig. 1). Of the core members of the care team, only the hematologists were supported with < 50% 340B program income at the majority of the HTCs and were primarily funded by billing to third party payers. The supporting services of genetic counselors were funded at a > 90% level by 5 of 13 HTCs that reported providing this service at comprehensive care visits.

In 2013, the approximate numbers of annual encounters with core members of the care team funded by 340B during comprehensive care visits were 10,092 encounters by nurses, 9048 encounters by hematologists, 9048 encounters by social workers, and 7656 encounters by physical therapists within participating HTCs. The 340B program income support for clinical staff salaries at outpatient and follow-up visits is similar to that for the comprehensive care visits. Most centers fund > 50% of the salary costs of non-physician staff utilizing 340B program income.

### Care Coordination and Case Management Activities

**Fig. 3:**
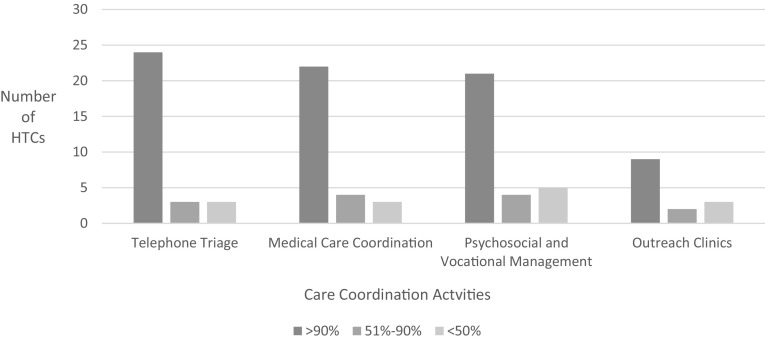
Number of centers and proportion of care coordination activities funded by 340B. 31 of 31 HTCs responded to these questions

Twenty-nine centers reported providing 57,072 urgent or emergent telephone triage encounters and 62,640 medical care coordination encounters, almost all non-billable services. Thirty centers reported 28,880 encounters for psychosocial and vocational services, almost all non-billable services. Figure 3 demonstrates that the majority of centers support staff salaries for coordination of care and case management services with 340B income at > 90% level (Fig. 2).

### Community Education and Support Activities

**Fig. 4:**
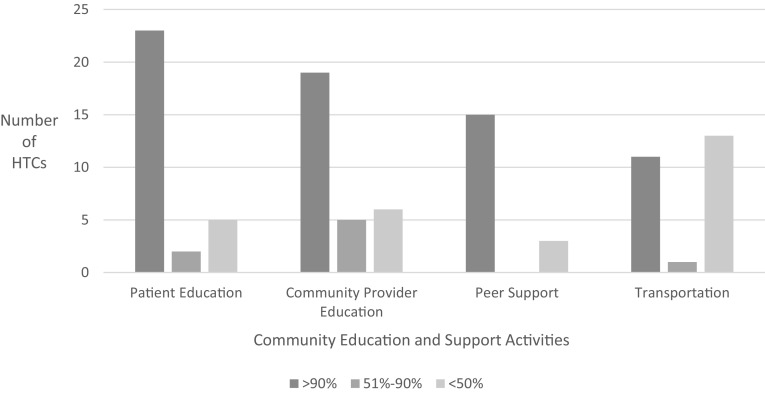
Number of HTCs providing community education and support activities and percentage of activities funded by 340B. 31 of 31 HTCs responded to these questions

HTCs provide education for patients and community providers; peer support groups for men, women, young families, peers, parent groups, and patients with thrombosis; and financial assistance with transportation (Fig. 4). The majority of HTCs support these activities with > 90% of staff time funded through 340B program income. During 2013, educational services were provided to 15,480 patients and families and 2160 community providers. Over 3000 patients at 25 centers received financial assistance for transportation, with almost half the centers reporting > 90% funding from 340B program income to help patients access care. Staff salaries for home and school visits by the HTC team are almost entirely supported by 340B program income.

### Camps

In 2013, 20 of the 31 HTC respondents offered 26 overnight camp programs serving 1170 children and adolescents. Nine centers sponsored 16 family camps with 624 individuals attending. Providers most frequently attending the overnight camps included nurses (n = 19), hematologists (n = 15), social workers (n = 14), physical therapists (n = 13), and nurse practitioners (n = 11). Staff attendance at camps is rarely a billable service. Approximately half of the centers support salaries for staff attending camps at the > 90% level and half support camps at ≤ 50% level.

## Discussion

The standard of care for children with special health care needs is to provide a multidisciplinary care team (Pai et al. 2016). Over the years, funding for the HTCs’ care model, a model providing services to individuals with rare, expensive, inherited disorders, has increasingly relied on income from the 340B program. Generally, only the physician time is reimbursable by insurance during the annual comprehensive and outpatient visits, omitting services of nurses, social workers, and physical therapists who provide an integrated and coordinated approach consisting of assessment, treatment and support for self-management. Most survey respondents indicated that the 340B program supported a significant percentage of salary for social workers, nurses and physical therapists.

Beyond providing assessments at comprehensive care centers, core staff spend an extensive amount of time on telephone triage and medical care coordination. Results indicate that staff engaged in approximately 57,072 urgent or emergent telephone triage encounters and 62,540 medical care coordination encounters in 2013, the majority of which are not billable. This ongoing integrated care management is critical to prevent complications that can lead to progressive joint disease, disability, and loss of income for the affected individual, as well as increased medical and social costs to society. Access to specialized care is challenging for many patients as many live far from the HTCs (Saxena 2013; Zhou et al. 2011). To maintain comprehensive and integrated care, the core staff at the HTCs coordinate care with local providers, emergency room specialists, and other specialists who lack familiarity with treating bleeding disorders. Providing these essential services may not be sustainable without 340B program income.

The HTCs begin to educate children in self-management at an early age (Cassis 2007). Anecdotally, patients and providers attribute their success in learning self-infusion and transition to medical independence to peer education and support (Sterling et al. 2012). HTCs conduct community outreach through home visits and school visits to provide education on self-management within the patient’s community and foster self-management. The 340B program supported 4832 home and school visits, a critical component of patients’ coordination of care with outside providers. Many HTCs offer camps for children and families with bleeding disorders to provide a peer environment outside the clinic through which children learn and are supported from older children on how to manage their care, including self-infusion. Both child and family camps are offered with the support of 340B program income funding in addition to other funding support such as National Hemophilia Foundation chapter support and pharmaceutical financial assistance.

Hemophilia and VWD are rare blood disorders and persons affected by these disorders are geographically dispersed throughout the United States. Many patients reside hundreds of miles from the nearest HTC. To improve access to care, HTCs have established outreach clinics where the HTC staff offer comprehensive clinics at locations more accessible to the rural population. These clinics save the patients both travel time and costs. Patients with limited financial resources may not be able to access comprehensive care clinics without the 340B program income support for these outreach efforts.

### Survey Limitations

The national 2012 Hemophilia Data Set (HDS), a national registry of HTC patients, reports that the median size of HTC patient populations is 200 patients (Forsberg 2010). This survey sample is over-represented by HTCs caring for large populations of patients. Many of the smaller centers with less established 340B programs use their limited 340B program funds solely to fund the salaries of core staff. Larger centers with more established 340B programs are able to offer more extensive services that are not reimbursed. As the purpose of this survey was to assess the broader spectrum of non-reimbursable services funded by 340B program income, the survey over-represents the larger, more established programs. However, the total estimated number of patients served by centers responding to this survey is 10,000. Therefore, the results of this survey represent care received by approximately one-third of the HTC population.

Another limitation was the inability to collect patient specific data from HTCs. This data is proprietary and participants would only submit data on the ranges of the numbers of patients served. Furthermore, the survey collected information on ranges of funding support instead of exact amounts. The staff effort required to collect the actual dollar amount of 340B program income funding for each service was beyond the scope of this survey.

## Conclusion

The results from this survey of 31 centers with established programs demonstrates the HTCs’ reliance on 340B program support for vital comprehensive services, that are otherwise non-reimbursable, and highlights the importance of the 340B program in sustaining the high quality of care and in increasing access for a geographically dispersed, medically vulnerable population. The 31 centers represent over 10,000 patients which is approximately one-third of the overall population of the US HTCs. The 340B program income in the calendar year 2013 supported over 200,000 patient and family encounters at 31 HTCs across the US including comprehensive care visits; outpatient and other follow-up visits; coordination of care; patient and family education and support; camp services; and outreach clinics and telemedicine. This survey demonstrates that many of the HTC services that are critical to providing optimal care and preventing complications are not reimbursed by third-party payers. HTCs are dependent on 340B program income to sustain high quality, accessible, comprehensive care for a medically vulnerable and complex patient population. It is unlikely this evidence-based model could be sustainable without 340B program income. Future data collection efforts should focus on an assessment of the utilization of 340B program income at smaller centers with new 340B programs and trends in the level of services supported by 340B program income over time.
